# Predicting GNSS Availability in Urban Canyons: A Scalable Automated 3D Ray-Tracing Approach Integrating OpenStreetMap and Terrain Elevation Data

**DOI:** 10.3390/s26196313

**Published:** 2026-10-06

**Authors:** Andrea Maffia, Tiziano Cosso, Giorgio Delzanno

**Affiliations:** 1 DIBRIS—Department of Informatics, Bioengineering, Robotics and Systems Engineering, University of Genoa, 16146 Genoa, Italy; 2 Gter s.r.l., 16128 Genova, Italy

**Keywords:** GNSS, urban canyons, ray tracing, NLOS, satellite visibility, intelligent transportation systems, autonomous driving

## Abstract

Continuous and reliable GNSS positioning is a requirement for autonomous driving and Intelligent Transportation Systems. While techniques such as RTK provide centimeter-level accuracy in open-sky conditions, their performance severely degrades in urban canyons due to Non-Line-of-Sight (NLOS) reception. Traditional autonomous routing algorithms primarily optimize travel time, disregarding GNSS signal quality, which can lead vehicles into areas with critical positioning difficulties. To address this gap, this paper presents a highly scalable, automated 3D ray-tracing framework designed to predict GNSS signal availability across urban environments. Many urban models rely on extruded building footprints assuming flat terrain; the proposed methodology integrates 3D building geometries from OpenStreetMap with a Digital Elevation Model (DEM). This approach better accounts for both artificial structures and natural topographic occlusions. By calculating the mean number of Line-of-Sight (LOS) satellites and Dilution of Precision (DOP), the framework generates a map of GNSS signal quality, providing a geometric upper bound of signal availability because diffraction is not modeled. The system is evaluated across Genoa, Italy, and validated against the KLT (Kowloon Tong) Dataset collected in Hong Kong; with the latter, the model achieves an NLOS classification precision of 95.0% and a LOS recall of 99.2%.

## 1. Introduction

### 1.1. Motivation

Continuous and reliable positioning is a fundamental requirement for autonomous driving and Intelligent Transportation Systems (ITS) [1]. Global Navigation Satellite Systems (GNSSs) generally provide satisfactory positioning performance in open-sky environments. However, their availability and accuracy can deteriorate significantly in urban canyons, where tall buildings, narrow streets, and complex terrain obstruct or reflect satellite signals [2,3]. These phenomena may cause signal blockage, multipath propagation, and Non-Line-of-Sight (NLOS) reception, thereby reducing the integrity and continuity of GNSS-based positioning.

Three-dimensional mapping-aided (3DMA) GNSS techniques can mitigate these limitations by exploiting information about the surrounding environment. In particular, three-dimensional ray-tracing methods can predict satellite visibility and signal obstruction at candidate receiver locations [4,5]. This information can support several applications, including GNSS performance assessment, localization algorithms, deployment planning, and GNSS-aware routing.

GNSS-aware routing extends conventional route planning by considering expected satellite visibility or positioning quality in addition to standard criteria such as distance and travel time. This capability is particularly relevant for autonomous and safety-critical vehicles, which may benefit from selecting routes that provide more reliable positioning conditions.

The construction of accurate and scalable visibility models remains challenging. Urban-scale simulations must jointly account for artificial structures, natural terrain, heterogeneous geospatial datasets, coordinate reference systems, and a potentially very large number of receiver–satellite visibility queries. Consequently, a practical framework must balance geometric accuracy, computational efficiency, automation, and scalability.

### 1.2. Technical Contributions

This paper presents an automated and scalable three-dimensional ray-tracing framework for assessing GNSS satellite visibility over large urban and non-planar environments. Unlike approaches based exclusively on building footprints and flat-ground assumptions, the proposed methodology integrates three-dimensional building models with Digital Elevation Models (DEMs). It can therefore represent signal occlusions caused by both artificial structures and natural terrain.

The main technical contributions of the work are as follows:

- Integrated building and terrain visibility modeling: The framework combines extruded three-dimensional building geometries with elevation data to model both local building obstruction and terrain-induced satellite masking.
- Automated acquisition and harmonization of geospatial data: A processing pipeline retrieves and prepares building information from OpenStreetMap and elevation data from Copernicus, reducing the manual effort required to construct the simulation environment.
- Consistent spatial registration: The framework aligns geospatial data, candidate receiver locations, and satellite directions within a common coordinate representation. This step addresses inconsistencies between projected map coordinates and the geodetic reference frames used for satellite positioning, which may otherwise introduce systematic visibility errors [6].
- Efficient ray–geometry intersection testing: A Bounding Volume Hierarchy (BVH) [7] is employed to accelerate intersection queries between satellite rays and three-dimensional building geometry, improving computational scalability when processing large numbers of receiver locations and satellite configurations. At each BVH leaf node, exact ray–triangle intersections are evaluated using the Möller–Trumbore algorithm in barycentric form [8].
- Hierarchical treatment of signal obstruction: Terrain masking and building obstruction are evaluated through complementary procedures, enabling satellite–receiver links to be classified according to their visibility conditions.
- Support for city-scale GNSS availability assessment: The resulting visibility information can be aggregated into spatial indicators suitable for the construction of GNSS availability maps and for the definition of GNSS-aware routing strategies.

Overall, the proposed framework transforms heterogeneous geospatial and satellite data into actionable information about expected GNSS availability while limiting the computational cost associated with large-scale three-dimensional visibility analysis.

### 1.3. Plan of the Paper

The remainder of the paper is organized as follows. the current section reviews existing 3DMA GNSS techniques, ray-tracing urban models, terrain integration, and spatial acceleration algorithms. Section 2 details the automated acquisition and integration of multi-source geospatial data (OSM and DEM), the road network sampling scheme, the terrain horizon pre-filtering, the CPU-built BVH hierarchy, and the batched GPU-accelerated ray-tracing traversal kernel. Section 3 presents the experimental evaluation, including the macro-scale spatiotemporal satellite availability maps generated over Genoa, Italy, the statistical characterization of DOP, the empirical validation against the open-source KLT dataset [9] collected in Hong Kong, and a quantitative assessment of the framework’s computational performance and throughput. Section 4 critically contextualizes the scientific boundaries, physical interpretations, and assumptions of the deterministic geometric approach. Finally, Section 5 summarizes the main findings and outlines directions for future research.

### 1.4. Related Work

Three-dimensional environmental models have increasingly been used to characterize GNSS propagation in dense urban environments. Existing 3DMA GNSS approaches exploit building geometry to predict satellite visibility, identify NLOS signals, estimate multipath conditions, or improve positioning through shadow matching and related map-aided techniques [2,3,4,5].

Early visibility and ray-tracing methods frequently relied on two-dimensional building footprints, simplified extrusion rules, or flat-ground assumptions [2,10]. These representations reduce model-construction and computational costs and may provide adequate approximations in relatively flat urban areas. However, they do not explicitly capture the effect of terrain elevation, slopes, hills, and other non-planar features that can modify the local horizon and obstruct low-elevation satellite signals [11,12]. Their applicability may therefore be limited in cities and road networks characterized by substantial topographical variability.

More detailed ray-tracing approaches employ high-resolution three-dimensional city models to represent building façades, roofs, and other urban structures. Although such models can improve the realism of propagation analysis, their construction and processing are computationally demanding. Their use at city scale may require extensive pre-processing, large memory resources, and efficient spatial acceleration techniques. In addition, high-fidelity models are not consistently available across different geographical areas, which limits the portability and reproducibility of approaches that depend on proprietary or manually prepared datasets.

Terrain-aware GNSS studies commonly estimate the local horizon from DEMs or other elevation products [11,12]. These methods can efficiently determine whether a satellite is hidden by surrounding terrain, but they generally do not represent fine-grained urban obstruction caused by individual buildings. Conversely, building-centered 3DMA methods often provide detailed local obstruction information while neglecting terrain or treating the ground as a planar surface. Integrating the two forms of obstruction is therefore necessary to obtain a more complete representation of satellite visibility in geographically complex urban environments. A Bounding Volume Hierarchy (BVH) is employed to accelerate ray–geometry intersection queries in [7]. Exact ray–triangle intersections are evaluated using the Möller–Trumbore algorithm [8].

Another important issue concerns the integration of heterogeneous spatial data. Building models, terrain products, receiver locations, and satellite states may be expressed in different projected, geodetic, or Earth-centered coordinate systems. Inaccurate transformations or inconsistent reference frames can misalign rays and environmental geometry, producing systematic errors in visibility classification [6]. A robust simulation workflow must consequently make coordinate transformation and spatial registration explicit components of the methodology.

Finally, the scalability of ray-tracing analysis depends on the efficiency of spatial intersection queries. Testing each receiver–satellite ray against every geometric primitive becomes impractical as the number of candidate receiver locations, epochs, satellites, and building surfaces increases. Spatial indexing and hierarchical acceleration structures, including BVHs, can substantially reduce the number of required geometric tests and are therefore particularly relevant to large-scale GNSS visibility assessment.

Related approaches have combined particle filtering with 3D urban models [13], integrated shadow matching with 3D-mapping-aided GNSS ranging [14], and applied factor graph optimization to robust urban GNSS positioning  [15].

The approach proposed in this paper addresses these limitations through a unified pipeline that combines building geometry and terrain elevation, explicitly manages their spatial registration, automates the acquisition and preparation of openly available geo-spatial data, and employs BVH-based acceleration for large numbers of visibility queries. Its primary objective is not high-fidelity electromagnetic propagation modeling, but an efficient and geographically comprehensive classification of satellite visibility suitable for GNSS availability mapping and routing applications.

## 2. Materials and Methods

This section delineates the integration of multi-source geospatial data and the implementation of the ray-tracing algorithm used to characterize satellite visibility. Specifically, we used a high-throughput, GPU-accelerated software framework utilizing NVIDIA CUDA to execute massively parallel ray-tracing simulations, adapted from the open-source gnss_gpu library [16], which is essential for overcoming the computational bottlenecks inherent in analyzing complex, high-resolution urban environments.

**Figure 1:**
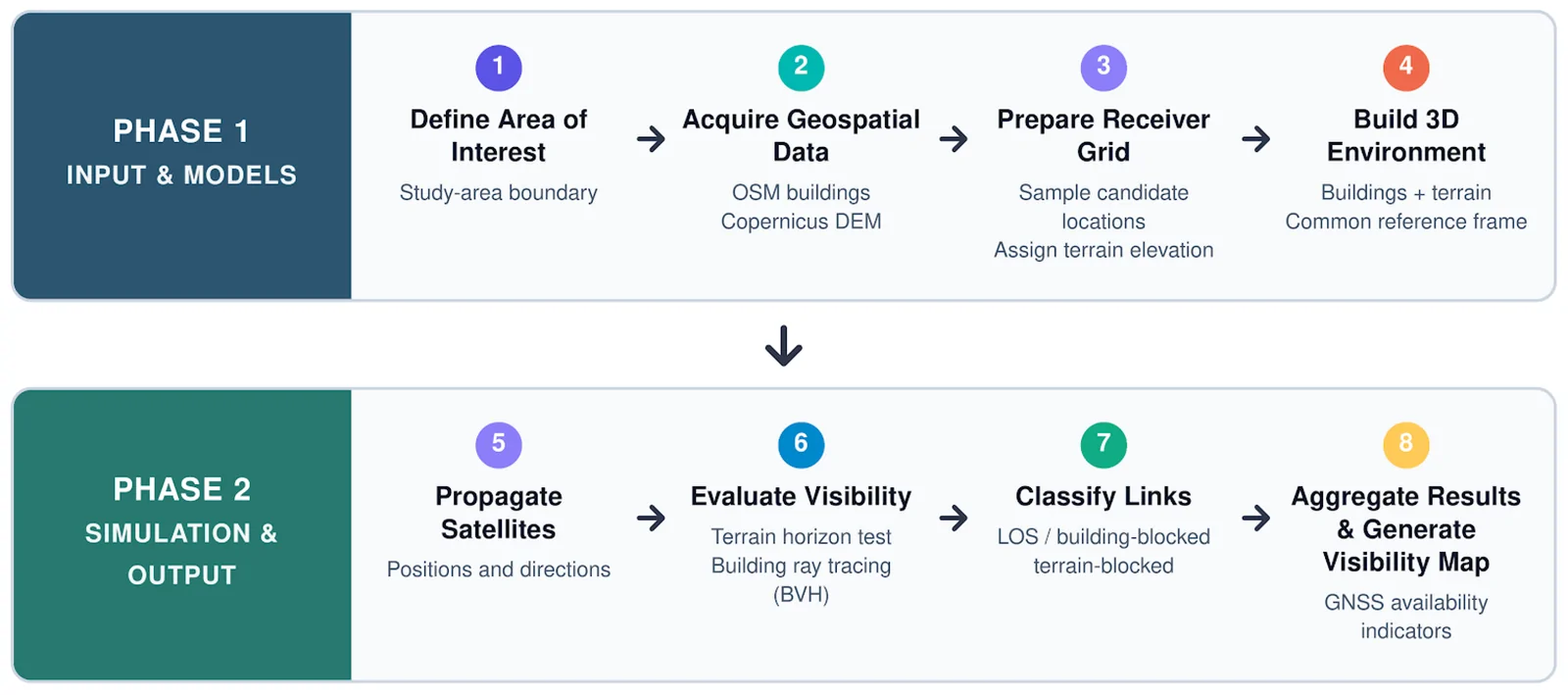
Architectural workflow and data pipeline of the proposed automated GNSS availability framework.

The end-to-end pipeline in Figure 1 streamlines the automated acquisition of building geometries from OpenStreetMap and Digital Elevation Models, enabling precise, automated anchoring of these structural elements directly onto the terrain mesh (steps 1–4).

For any specified temporal epoch, the software dynamically retrieves precise satellite ephemeris data and performs a granular propagation analysis along street segments, sampled at 1m intervals; this high-density sampling ensures the detection of rapid, localized changes in visibility conditions caused by complex urban geometries (steps 5–8). Through this intensive analysis, the system calculates definitive Line-of-Sight and Non-Line-of-Sight conditions, as well as the resulting Dilution of Precision for all visible satellite constellations, thereby providing the necessary fidelity to identify navigation risks in challenging urban environments.

### 2.1. Geospatial Data Sources

The geometric representation of the simulated environment is constructed from two complementary open geospatial data sources. OpenStreetMap (OSM) provides the vector data used to extract the road network and building footprints within the area of interest [17,18]. Road geometries are employed to generate the candidate receiver locations, whereas building polygons are converted into three-dimensional obstacle meshes using the available height-related attributes. In particular, explicit building heights are used when available; otherwise, heights are estimated from the number of building levels or assigned a predefined fallback value. Since OSM is a collaboratively maintained database, the availability and accuracy of geometric and semantic attributes may vary spatially.

Terrain elevation is obtained from the Copernicus Digital Elevation Model (Copernicus DEM) [19]. The GLO-30 product, distributed as georeferenced raster tiles with a grid spacing of 1 arc second (approximately 30m at the Equator), is used to assign the receiver altitude and derive the local terrain-horizon profiles. Although commonly referred to as a DEM, the Copernicus product is technically a digital surface model derived from the edited WorldDEM dataset and therefore represents the Earth’s surface, including vegetation, buildings, and other above-ground structures. Before integration, OSM and Copernicus DEM data are clipped to the area of interest and transformed into a common projected coordinate reference system to ensure consistent metric distance, height, and ray-direction computations.

### 2.2. Area Definition and Road Network Sampling

The input to the processing pipeline is an Area of Interest (AOI), specified as a rectangular latitude–longitude bounding box.

**Figure 2:**
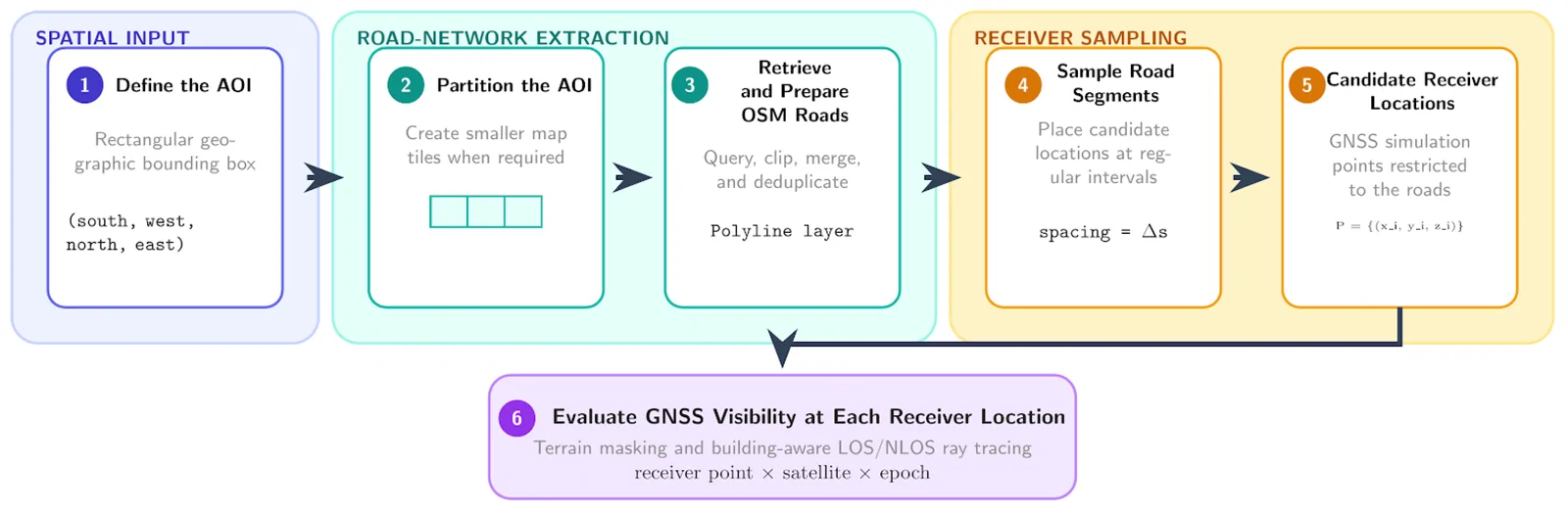
Steps to pass from an AOI to the actual input to the ray tracing algorithm.

**Figure 3:**
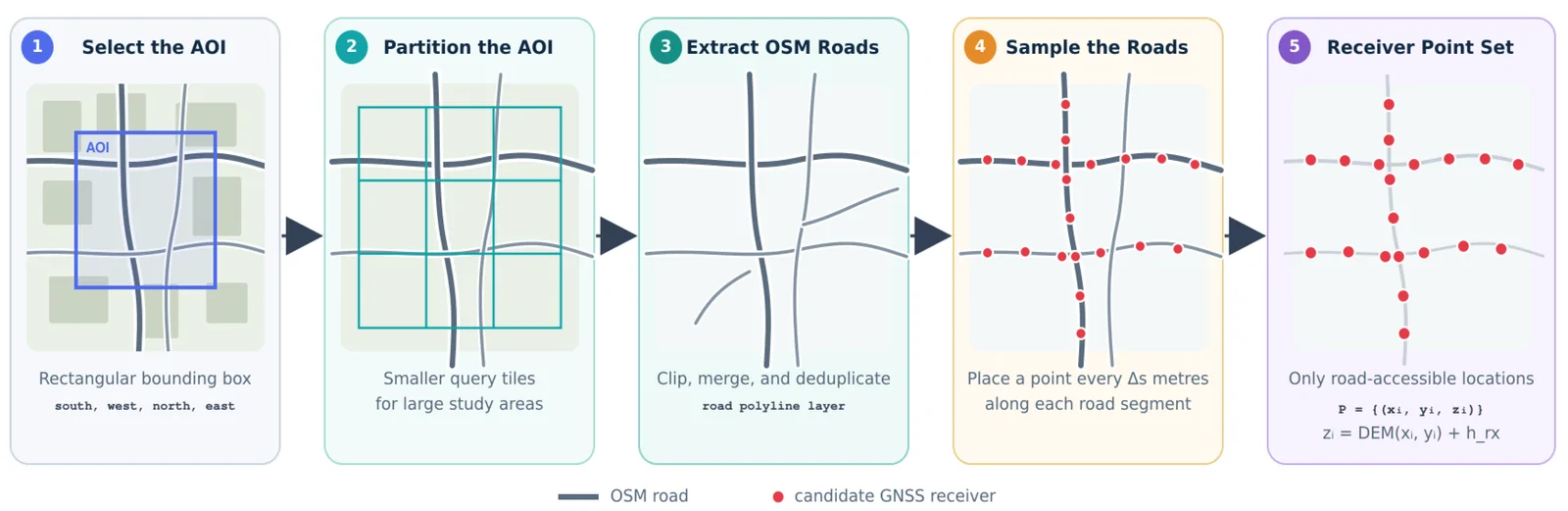
Example of grid extraction.

The preparation pipeline that transforms an AOI into the data structure used in the rest of the computation is shown in Algorithm 1 (Figure 2 and Figure 3).

Because the analysis is intended for mobile or pedestrian navigation scenarios, the receiver domain is not treated as a continuous surface; instead, it is strictly constrained to the local road network. This approach produces a physically meaningful sampling space and avoids evaluating mathematically valid but practically inaccessible locations (e.g., inside building volumes or unnavigable terrain).

Within the selected area, the road network is retrieved from OSM. Each road polyline is discretized into a set of receiver candidate points at approximately uniform spatial spacing, parameterized at exactly one meter intervals along the street centerlines, referred to as $s_{\mathrm{step}}$. This high-density spatial discretization ensures the detection of rapid, localized changes in visibility conditions.

For large-scale geographic domains, spatial tiling is employed during the data acquisition phase to ensure I/O stability and memory efficiency when querying OpenStreetMap APIs. The target bounding box is decomposed into a regular grid of approximately square latitude–longitude tiles parameterized by a side length $s_{\mathrm{tile}}$ as shown in Algorithm 1. Upon retrieval, sampled road centerlines across all tiles are merged and deduplicated to yield a unified receiver point dataset.

It should be noted that this spatial tiling serves as a regular geometric decomposition for data handling rather than an adaptive computational load-balancing mechanism. During GPU simulation, computational load balancing is handled via uniform tensor batching across receiver-point chunks and temporal epoch blocks.
**Algorithm 1** Road-network receiver sampling**Require:** Study-area bounding box B(coordinates)**Require:** Optional tile size $s_{\mathrm{tile}}$$s_{\mathrm{tile}}\leq 0$ disables tiling**Require:** Along-track sampling step $s_{\mathrm{step}}$▹ e.g., 25m**Ensure:** Set P of unique on-road GNSS receiver candidates  1: **procedure** SampleReceivers($B,s_{\mathrm{tile}},s_{\mathrm{step}}$)  2:       
G
←
SplitIntoTiles
$\left(B,s_{\mathrm{tile}}\right)$ ▹ Use {B} when tiling is disabled  3:       P←∅  4:       **for all** tiles τ∈G **do**  5:             R
←
FetchOSMHighways
(τ) ▹ OpenStreetMap highway layer  6:             **for all** road polylines ℓ∈R **do**  7:                  P←P ∪
SampleAlongPolyline
$\left(\ell ,s_{\mathrm{step}}\right)$
  8:             **end for**
  9:        **end for**
10:       **return** 
Deduplicate
(P)▹ Retain unique on-road GNSS candidates11: **end procedure**

Computational Complexity of Algorithm 1.

Let *L* denote the total length of OSM highway polylines inside the studi Bounding-box *B*, and let $s_{\mathrm{step}}$ be the along-track spacing. Sampling points every $s_{\mathrm{step}}$ metres along the road network produces P = $O(L/s_{\mathrm{step}})$ receiver candidates and costs $O(L/s_{\mathrm{step}})$ time, plus O(P) expected time for de-duplication.

### 2.3. Urban Obstruction Model and Terrain Representation

**Figure 4:**
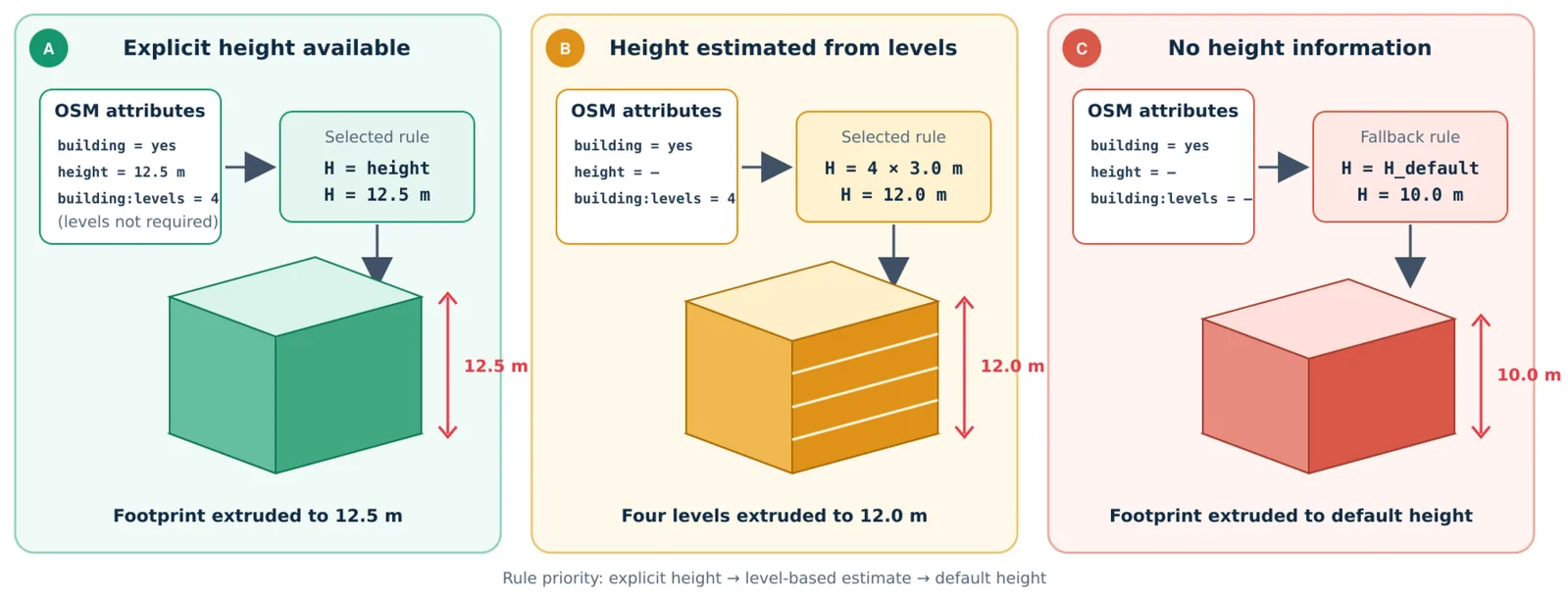
From OSM attributes to building extrusion.

A three-dimensional obstruction model is generated from OSM building footprints. Each building polygon is converted into a volumetric mesh by vertical extrusion, utilizing LoD1 models with either explicit height attributes or inferred structural metadata. The construction of the map and sampling of the roads has been adapted from the open-source map3d library [20]. Missing height metadata are resolved via an explicit fallback hierarchy as shown in Figure 4.

More specifically, the algorithm consists of the following steps:

- if the explicit height tag is absent, height is inferred from the building:levels attribute assuming 3.0 m per floor.
- if both attributes are missing, a conservative default height of 10.0 m is assigned

**Figure 5:**
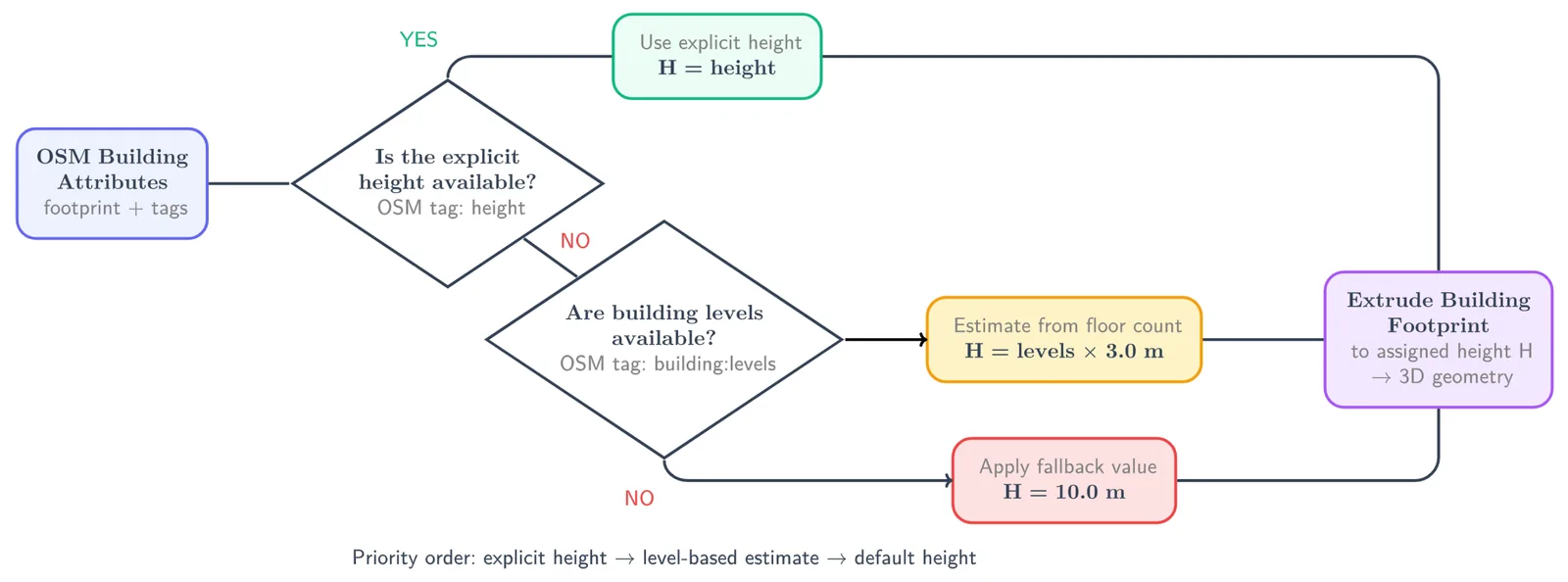
Workflow for building extrusion.

The workflow is shown in Figure 5.

**Figure 6:**
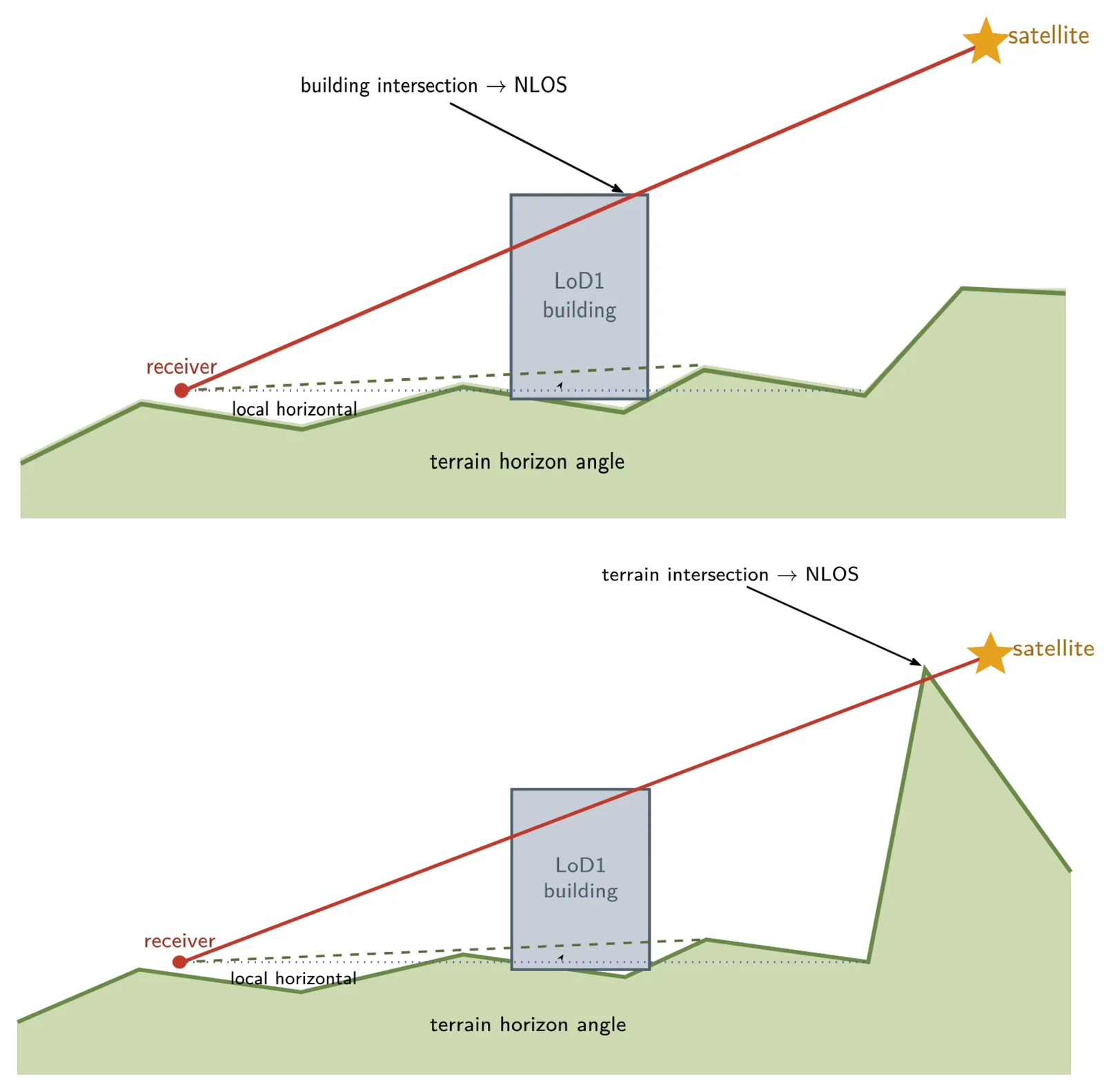
Visibility obstacles: buildings and terrain.

Terrain enters the pipeline in two distinct roles. First, it defines the receiver altitude. Each sampled road point is assigned to an elevation derived from a DEM, preventing vertical inconsistencies between roads and buildings. The base coordinates of the building vertices are anchored to the local terrain elevation f(x,y), and the roof structure is extruded along the gravity vector:

Second, the terrain acts as an additional visibility obstacle, see Figure 6. A terrain horizon profile is computed around each receiver location by sampling the DEM radially over azimuth and distance. This yields the minimum elevation angle above which a satellite must appear to remain visible over the surrounding relief, allowing the methodology to distinguish between obstruction by distant terrain and obstruction by immediate urban buildings.

The pseudocode for computing the building obstruction model is shown in Algorithm 2. The BuildBVH computes a Bounding Volume Hierarchy used to accelerate the interection step with rays as discussed later on in this section.
**Algorithm 2** Construction of the building obstruction model**Require:** OpenStreetMap building layer OSM**Require:** Study-area bounding box B(coordinates)**Require:** Default per-floor height $h_{\mathrm{floor}}$▹ default: 3.0m**Require:** Default building height $H_{\mathrm{def}}$
▹ default: 10.0m**Ensure:** Triangle mesh T of LoD1 building solids**Ensure:** Bounding-volume hierarchy BVH over T  1: **procedure** BuildObstructionModel($OSM,B,h_{\mathrm{floor}},H_{\mathrm{def}}$)  2:       T←∅  3:       **for all** building footprints b∈OSM∩B **do**  4:             H ←
InferBuildingHeight
$\left(b.\mathrm{tags},h_{\mathrm{floor}},H_{\mathrm{def}}\right)$ ▹height; else    
building:levels$\;\times \;h_{\mathrm{floor}}$; else $H_{\mathrm{def}}$  5:             **if** H≤0 **then**  6:                   **continue**  7:             **end if**  8:             T←T
∪
ExtrudeLoD1
(b,H)  9:       **end for**10:      
BVH ←
BuildBVH
(T)11:       **return** (T,BVH)12: **end procedure**

Computational Complexity of Algorithm 2.

Let N=|T| denote the number of triangles in the generated LoD1 building mesh. LoD1 extrusion produces this mesh in linear time O(N), i.e., proportional to the number of emitted building triangles. The subsequent SAH-based BVH construction has expected cost O(NlogN). Both stages are executed once per scene and do not depend on the number of visibility rays; they are therefore a one-time setup cost for the online LOS/NLOS queries.

### 2.4. Dynamic GNSS Simulation and Visibility Pre-Filtering

To capture the temporal variability of the satellite constellations, visibility is repeatedly sampled over a sequence of time epochs. At each epoch *t*, satellite positions $s_{i}\left(t\right)$ are computed from navigation ephemerides, propagated into Earth-Centered, Earth-Fixed (ECEF) coordinates, and then transformed into a local East-North-Up (ENU) coordinate frame to preserve numerical precision during vector operations.

Before performing computationally expensive building-ray intersection tests, a Visibility Pre-Filtering stage is executed. This stage computes the local elevation angle for each satellite and applies two rejection criteria Figure 6:

- Geometric Elevation Mask: Rejection of satellites below a standard operational threshold (e.g., $E_{min}=10{}^{\circ}$).
- Terrain Horizon Mask: Rejection of satellites falling below the radially sampled DEM profile.

Terrain horizon profiles are evaluated by radially sampling the DEM up to a maximum distance $R_{\max}$ of 20 km from each receiver location using a discrete spatial step of 60 m (333 sampling points per terrain evaluation ray). In the CPU profile implementation, an azimuthal discretization of 2° (180 angular bins) is used to build a pre-computed horizon cache. In the GPU-accelerated CUDA area-map pipeline, terrain visibility is evaluated by performing discrete 60 m radial sampling directly along each satellite’s true azimuth vector, avoiding fixed-grid interpolation artifacts. By filtering out satellites positioned below the terrain horizon or standard elevation mask early in the pipeline, the method avoids performing unnecessary 3D traversals and triangle-intersection queries for non-viable satellites.

The pseudocode of the terrain pre-filtering phase is shown in Algorithm 3.
**Algorithm 3** Geometric and terrain-based satellite visibility pre-filtering**Require:**
 Receiver position r**Require:**
 Satellite elevation and azimuth angles el and az**Require:**
 Digital elevation model *Z***Require:**
 Minimum elevation threshold $E_{\min}$**Require:**
 Maximum terrain-search radius $R_{\max}$**Require:**
 Radial sampling step Δr**Require:**
 Terrain-horizon angular margin $\delta_{h}$**Ensure:**
 Visibility class c∈{BELOW_MASK,TERRAIN_BLOCKED,CANDIDATE}**Ensure:**
 Terrain-horizon angle h(az)  1:**procedure** TerrainPrefilter($r,el,az,Z,E_{\min},R_{\max},\Delta r,\delta_{h}$)  2:      **if** $el<E_{\min}$ **then**  3:            **return** (BELOW_MASK,−π/2)  4:      **end if**  5:      h(az)←−π/2  6:      $z_{r}$ ← Altitude (r)  7:      **for** d←Δr **to** $R_{\max}$ **step** Δr **do**  8:            $r_{d}$ ← Offset (r,az,d)  9:            $z_{d}$ ← SampleDEM $\left(Z,r_{d}\right)$10:           $h\left(az\right)\leftarrow \max\left\{h\left(az\right),atan2\left(z_{d}-z_{r},d\right)\right\}$11:     **end for**12:     **if** $el<h\left(az\right)+\delta_{h}$ **then**13:           **return** (TERRAIN_BLOCKED,h(az))14:     **else**15:           **return** (CANDIDATE,h(az))16:     **end if**17:**end procedure**

Computational Complexity of Algorithm 3.

Let a *ray* denote one receiver–epoch–satellite visibility query, and let *R* be the total number of such queries processed in a campaign. Equivalently,

(1) R=PES,

where *P* is the number of receiver sample points, *E* the number of time epochs, and *S* the number of satellites evaluated at each epoch. Thus, *R* measures the size of the geometric workload.

For a single ray, Algorithm 3 first performs an O(1) elevation-mask check. If the satellite lies below $E_{\min}$, the procedure returns immediately. Otherwise, it marches radially along the satellite azimuth from Δr to $R_{\max}$ with step Δr. At each sample location, the DEM is queried. If the local terrain elevation angle exceeds the satellite elevation, the ray is flagged as occluded and the loop terminates immediately (early exit).

Consequently, the maximum number of radial steps is

(2) $$K=\left\lfloor \frac{R_{\max}}{\Delta r}\right\rfloor .$$

with the default parameters $R_{\max}=20\;\mathrm{km}$ and Δr=60m, the search requires at most K=333 DEM samples per ray.

While the terrain morphology is static for any given point *P*, computing the horizon on-the-fly for all *R* queries allows the framework to dynamically leverage GPU parallelization without the massive memory overhead of pre-computing and storing 3D horizon panoramas. Therefore, the *worst-case* per-ray cost is bounded by O(K), and the aggregate worst-case complexity over the entire simulation is

(3) $$O\;\left(R\cdot \frac{R_{\max}}{\Delta r}\right).$$

### 2.5. Bounding Volume Hierarchy (BVH) Acceleration

The computational bottleneck of area-based visibility analysis is evaluating whether the straight-line path between a receiver $x_{j}$ and a pre-filtered satellite $s_{i}\left(t\right)$ is obstructed by the urban 3D environment. A brute-force $O\left(N_{\mathrm{rays}}\times N_{\mathrm{facets}}\right)$ approach scales poorly. To make the simulation tractable, the pipeline integrates a Bounding Volume Hierarchy (BVH) [7] with GPU-parallel ray tracing.

#### 2.5.1. CPU-Based Construction with Surface Area Heuristic (SAH)

The hierarchy is built on the CPU before the simulation stage begins, capitalizing on the asymmetry that the static building mesh is reused across all temporal epochs. The BVH recursively partitions the triangle set into spatially coherent subsets, each enclosed by an Axis-Aligned Bounding Box (AABB).

**Figure 7:**
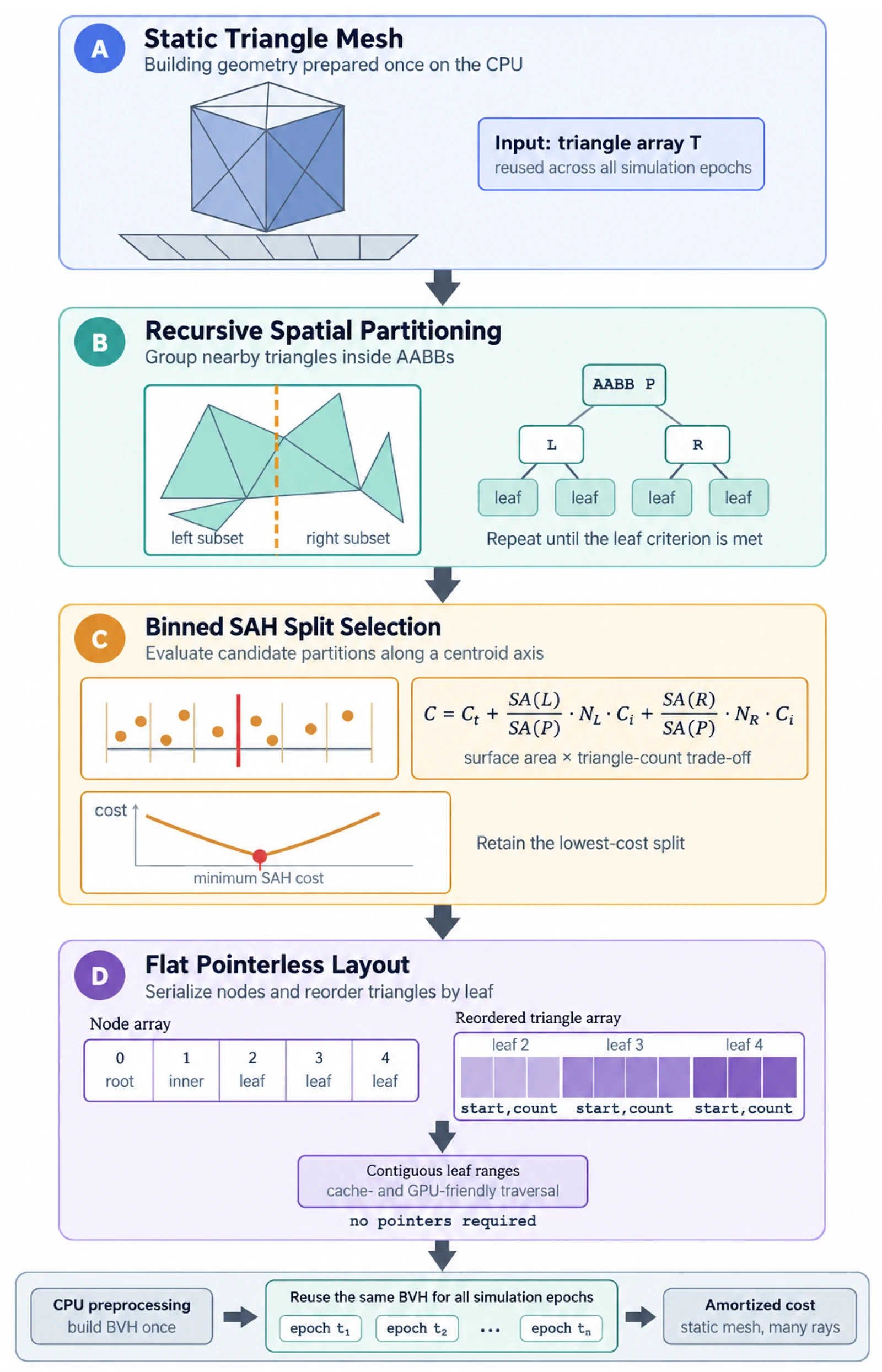
Construction of the BVH.

The split is selected using the Surface Area Heuristic (SAH), which aims to minimize the expected ray traversal cost by balancing subtree overlap and triangle count. A coarse binned SAH stage estimates a promising split direction, and candidate partitions are evaluated along the centroid axis. The split with the minimum SAH cost is retained. The resulting hierarchy is stored as a flat, pointer less node array. Triangles are then reordered according to the leaf ordering induced by the BVH, ensuring that each leaf references a contiguous range of memory. The idea is illustrated in Figure 7.

**Figure 8:**
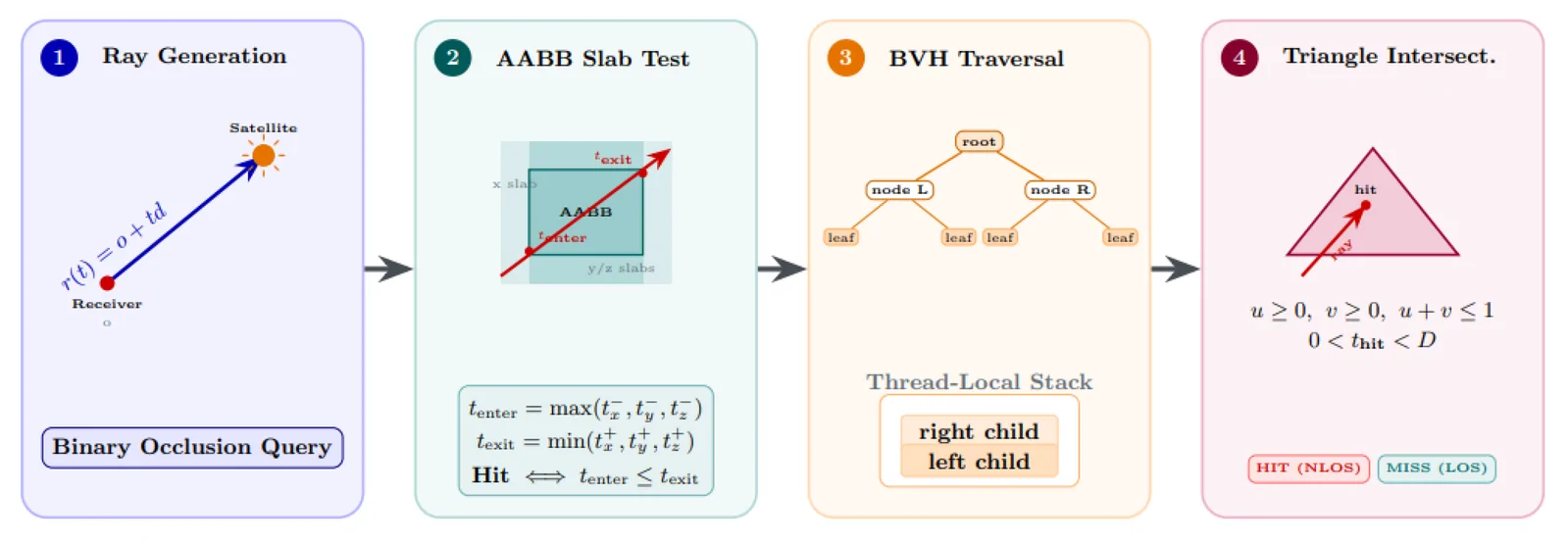
LOS/NLOS workflow.

The workflow and pseudocode that describe the LOS/NLOS procedure is shown resp. in Algorithm 4 and Figure 8.
**Algorithm 4** Building-based LOS/NLOS classification using BVH traversal**Require:** Receiver position r**Require:** Satellite position s**Require:** Bounding-volume hierarchy BVH over the building triangle mesh**Require:** Numerical tolerance ε>0**Ensure:** Boolean label
losBldg ∈
{
True,
False
}
**Ensure:** True if the segment $\bar{\mathrm{rs}}$ does not intersect any building triangle  1: **procedure** BuildingLOS(r,s,BVH,ε)   2:      d←∥s−r∥  3:       **if** d<ε **then**  4:             **return** True
▹ degenerate/coincident positions  5:       **end if**  6:       $\hat{u}\leftarrow \frac{s-r}{d}$  7:       stack ← [Root(BVH)]
  8:       **while** stack≠∅ **do**  9:             node ← Pop(stack)10:             **if** 
RayMissesAABB
$\left(r,\hat{u},\mathrm{AABB}\left(\mathrm{node}\right),\left[0,d\right]\right)$ **then**11:                    **continue**12:             **end if**13:             **if** 
IsLeaf
(node)
 **then**14:                    **for all**  T ∈ Triangles
(node) **do**15:                           (hit,*t*) ← MollerTrumbore
$\left(r,\hat{u},T\right)$16:                           **if** hit∧ε<t∧t<d **then**17:                                  **return** False
▹ early-exit NLOS18:                           **end if**19:                    **end for**20:             **else**21:                   
children ← Children(node)22:                    Push
(stack, children)23:             **end if**24:       **end while**25:       **return** True
▹ LOS26: **end procedure**

Computational Complexity of Algorithm 4.

Algorithm 4 decides building LOS/NLOS for one receiver–satellite pair by traversing a BVH over a triangle mesh of size *N*. Each visited node is tested against the ray segment $\bar{\mathrm{rs}}$ in O(1) time via a slab-based AABB test; leaf nodes apply the Möller–Trumbore intersection to a constant-bounded number of triangles.

Because the query stops at the first valid hit with ε<t<d, the expected cost for a well-built BVH is O(logN) per ray. In the worst case (e.g., a clear LOS ray that must exhaust all overlapping branches), the cost can reach O(N).

Over a campaign of R=PES rays, where P is the number of receiver sample points, E the number of time epochs, and S the number of satellites evaluated at each epoch. Thus, R measures the size of the geometric workload the aggregate complexity is therefore: O(RlogN) (expected), O(RN) (worst case). Thus, for typical urban meshes, building LOS scales linearly with the number of rays and only logarithmically with mesh size.

#### 2.5.2. Batched GPU Traversal and Early-Exit Ray Tracing

During simulation, ray tracing is executed on the GPU. The flattened BVH and reordered triangle arrays are copied to device memory. The framework organizes the simulation into epoch batches and points batches, where all visible candidate satellites are packed into contiguous arrays (tensors). A CUDA kernel evaluates the entire receiver–satellite batch in a single launch, amortizing kernel overhead and maximizing Streaming Multiprocessor (SM) occupancy.

For each receiver–satellite pair, the line-of-sight test is formulated as a ray–mesh intersection problem. The ray is parameterized as $r\left(\tau \right)=o+\tau \hat{d}$, with origin o at the receiver and unit direction $\hat{d}$ toward the satellite and is restricted to the segment $\tau \in \left[0,d_{sat}\right]$. BVH nodes are tested against this ray using a slab-based AABB intersection: for each coordinate axis x,y,z, the ray is clipped against the two parallel planes of the box, yielding an interval $\left[\tau_{\min},\tau_{\max}\right]$; the three axial intervals are then intersected, and the node is rejected if the resulting interval is empty or lies outside $\left[0,d_{sat}\right]$. To avoid repeated divisions, the reciprocal direction ${\hat{d}}^{-1}$ is precomputed once per ray. Hierarchy traversal is managed with an explicit stack in thread-local memory (registers): the root is pushed first, nodes whose AABB misses the ray are skipped, internal nodes push their children, and leaf nodes invoke early-exit Möller–Trumbore triangle tests. As soon as any triangle hit with $0<\tau <d_{sat}$ is found, the satellite is labeled NLOS and traversal terminates. At leaf nodes, exact triangle hits are tested with the Möller–Trumbore algorithm in barycentric form [8]. A hit is accepted only if the intersection distance lies strictly between the receiver and the satellite ($\varepsilon <\tau <d_{sat}$).

The LOS/NLOS classification is deterministic: a receiver–satellite path is assigned an NLOS state if a ray-triangle intersection occurs at a parametric distance $t<d_{\mathrm{sat}}$, where $d_{\mathrm{sat}}$ represents the Euclidean distance between the receiver and the satellite. To ensure floating-point robustness and prevent numerical boundary artifacts during Möller–Trumbore traversal, a standard tolerance of $\varepsilon =10^{-12}$ is applied. No tunable classification thresholds are utilized.

### 2.6. Spatio-Temporal Aggregation and Map Generation

The output of the batched CUDA evaluation is a Boolean matrix indicating the LOS/NLOS status for each valid receiver–satellite pair. These per-epoch results are accumulated over the full temporal window and converted into mean statistics for each sampled receiver location. The principal aggregated quantities include:

- Mean LOS Count: Satellites simultaneously visible and unobstructed by buildings.
- Mean NLOS Count: Satellites above the local visibility threshold but blocked by buildings.
- Mean Terrain-Blocked Count: Satellites excluded primarily by topography.
- Mean DOP: DOP obtained considering only the visible satellites

The final stage converts these tabulated statistics into an interactive thematic map. Each sampled road point is rendered as a colored marker whose color encodes a specific metric (e.g., mean LOS count), utilizing a continuous color scale.

### 2.7. Map Generation and Reproducibility

The final stage converts these tabulated statistics into an interactive thematic map.

The receiver table is joined to the sampled road-point geometry. A selected metric, such as mean LOS count, mean PDOP, or NLOS fraction, is encoded with a continuous color scale; fixed legend limits are used when multiple maps are compared.

Tooltips provide the receiver identifier, coordinates, metric value, valid-epoch count, and configuration identifier. Each export includes the OSM extraction date, DEM product and resolution, ephemeris source, coordinate and vertical reference systems, road filters, CPU/GPU terrain mode, and temporal window. These metadata distinguish temporal changes in satellite geometry from changes in input maps or numerical configuration.

## 3. Results

To validate the framework’s capabilities and computational efficiency, a series of simulations were generated across diverse urban environments of Genoa, Italy. This city serves as an ideal case study, characterized by distinct topographic constraints and a sharp contrast between its dense, medieval historic center and its open, unobstructed coastline.

### 3.1. Macro-Scale Satellite Visibility Analysis

**Figure 9:**
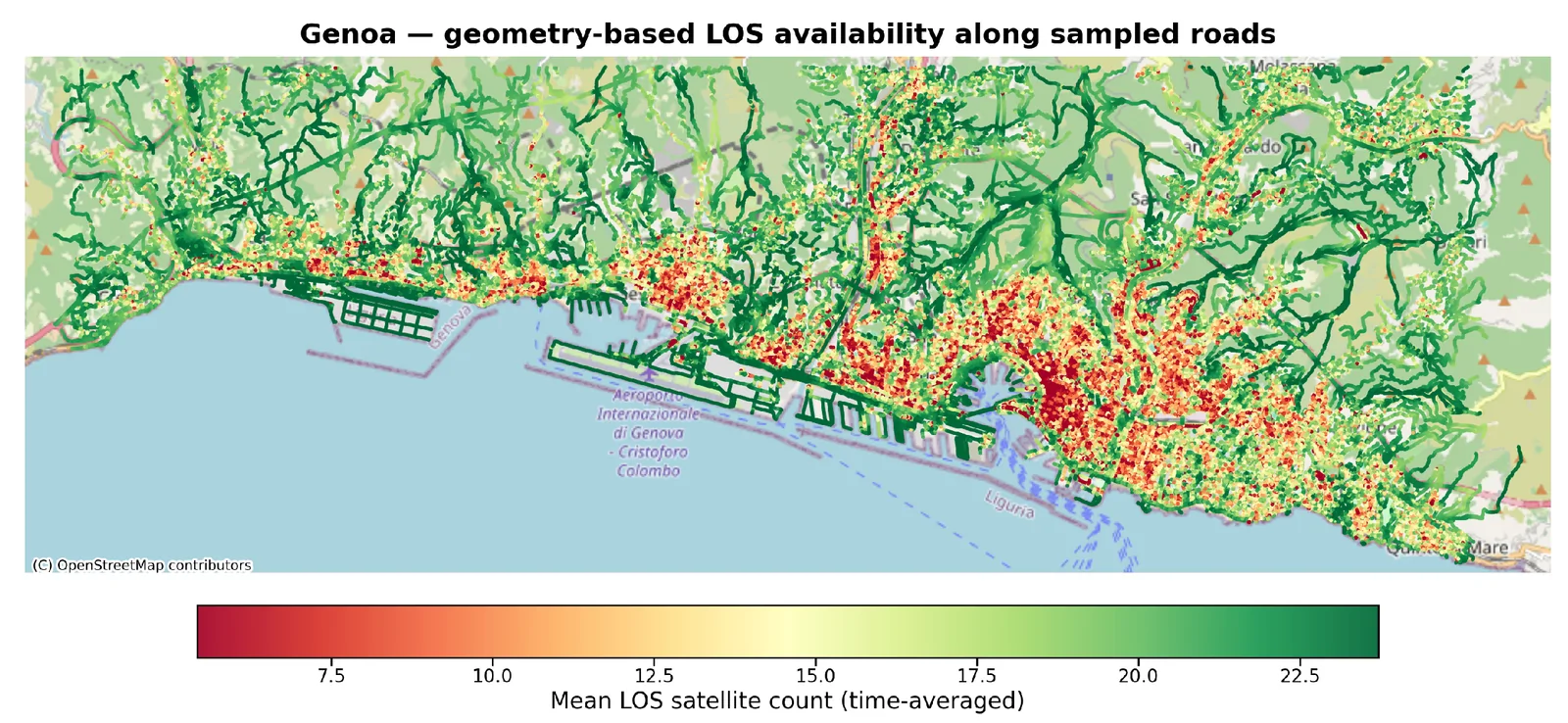
Macro-scale spatiotemporal thematic map displaying the time-averaged mean LOS satellite availability across the entire simulated road network of Genoa.

The primary objective of the macro-scale analysis is to map the spatial distribution of GNSS visibility across heterogeneous urban geometries. The simulation was conducted for a full 24-h period corresponding to 15 May 2026, utilizing orbit trajectories from the GPS, Galileo, and BeiDou constellations. Figure 9 displays the continuous spatiotemporal thematic map of average LOS satellite availability generated by our framework over the entire simulated road network of Genoa.

The spatial variations in satellite visibility directly mirror the geometric characteristics of the underlying built environment. Along the coastline and within the harbor infrastructure, the simulation registers optimal tracking conditions, with an average of LOS =20 satellites. In contrast, within the medieval historic center, the sky-view factor (SVF) drops severely due to the high aspect ratio of the narrow street canyons. In these regions, the average number of LOS satellites plummets to LOS = 9 satellites.

To check the capabilities of the ray-tracing core, we isolate two contrasting sub-scenarios extracted from the high-density simulation pipeline.

**Figure 10:**
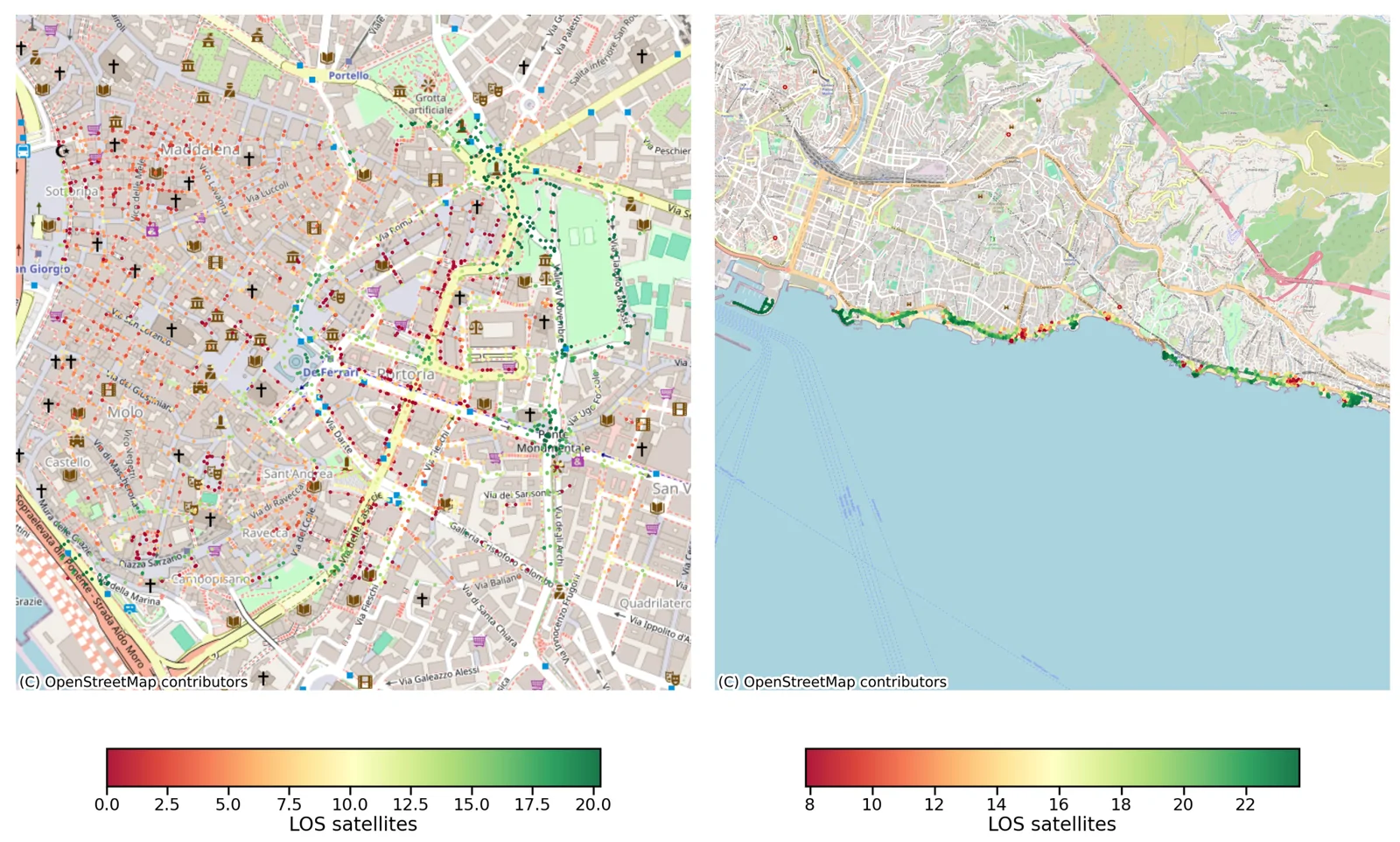
Historic center scenario, illustrating severe signal occlusion by high-aspect-ratio historical buildings (**left**); Coastal front scenario along the sea-facing boulevard, demonstrating an open horizon with near-optimal visibility (**right**).

In the Historic Center scenario of Figure 10 (left), the receiver is positioned along a narrow street segment bordered by historical buildings exceeding 15 m in height. The simulation illustrates how many satellites are systematically occluded by the building envelopes (triggering early-exit BVH hits). The signals that do reach the receiver are restricted to a narrow, near-zenith keyhole, which drastically distorts the geometric configuration of the visible constellation.

In the Coastal Front scenario of Figure 10 (right), the receiver is positioned along the sea-facing boulevard. Because the marine hemisphere is completely free of structural obstructions, the ray-tracing engine registers an open horizon. Satellites passing the initial terrain and geometric pre-filters remain in LOS, yielding a highly symmetric and distributed constellation geometry.

### 3.2. Statistical Characterization of Dilution of Precision (DOP)

Table 1 illustrates the statistical distribution of the HDOP values aggregated over a 24-h simulation cycle for both the historic core and the coastal area.

The statistical divergence is high. In the coastal zone, the HDOP distribution is highly concentrated near the theoretical ideal, with a mean of 1.42 ± 0.26 and a cumulative probability of almost 100% below an HDOP of 2.5. In the medieval historic center, a significant probability mass shifts toward critical regimes (mean HDOP of 4.52 ± 4.11).

### 3.3. Ablation Study: Impact of Terrain Horizon Modeling

To evaluate the specific contribution of the Digital Elevation Model (DEM) relative to 3D building occlusions, an ablation analysis was executed over 171,711 evaluation points across Genoa. Across the entire city, out of an average of ∼8.5 total obstructed satellite paths per epoch, terrain pre-filtering accounts for 1.21±0.68 blocked rays, meaning that 14.2% of all signal occlusions across the city are caused directly by terrain topography rather than building structures.

As detailed in Table 2, spatial disaggregation reveals strong morphological dependencies, as expected:

- Historic Center: Topographic masking accounts for less than 0.7% of total signal loss (<0.1 satellites per epoch), as high-aspect-ratio LoD1 building structures strictly dominate the local horizon.
- Coastal Zone: Terrain becomes the primary cause of signal loss, accounting for 51.3% of all obstructed satellite links (1.54 out of ∼3.0 blocked satellites per epoch). In open coastal environments, inland low-elevation satellite rays are masked almost exclusively by the surrounding mountain relief.

### 3.4. Validation of the Geometric LOS/NLOS Classifier

**Figure 11:**
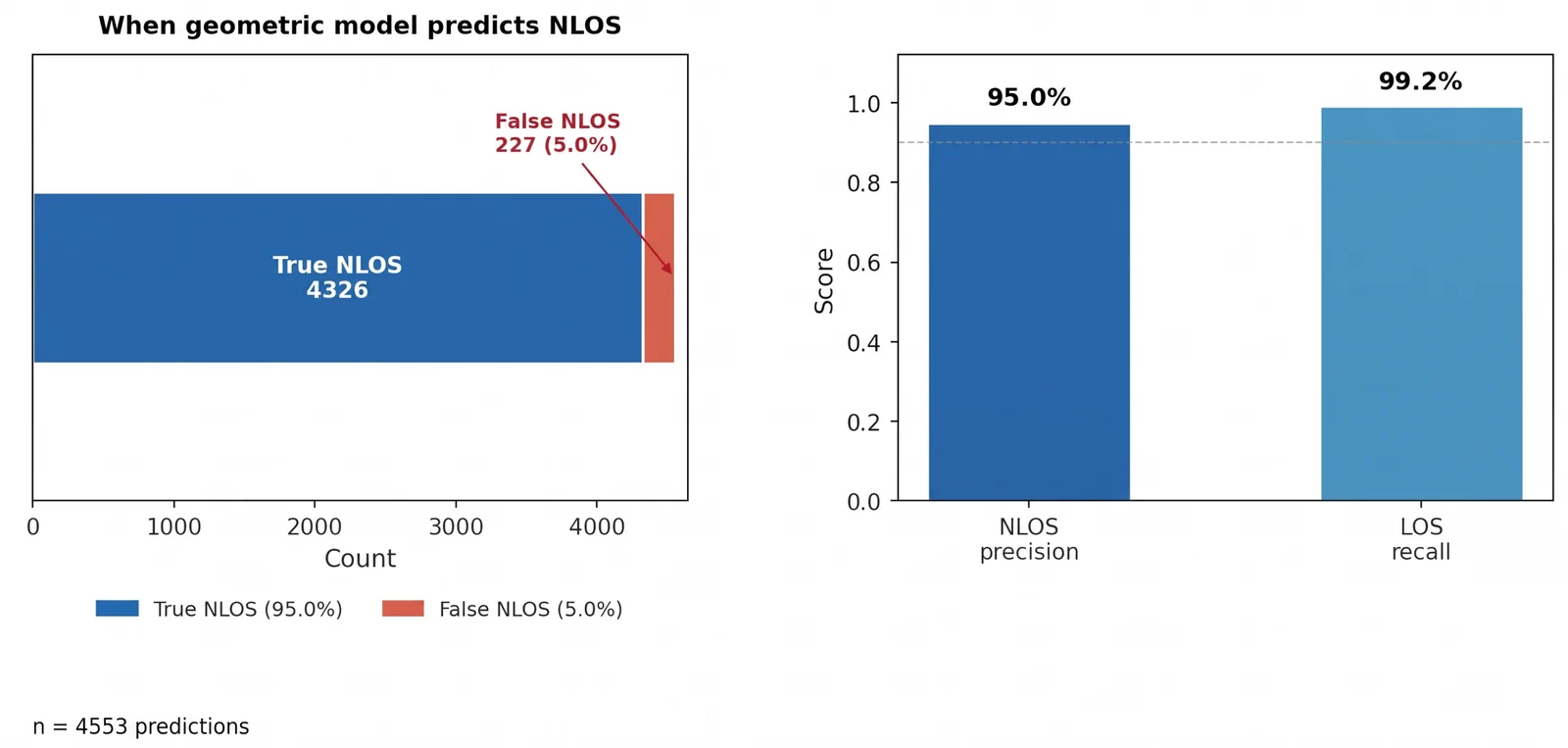
Statistics on when the framework predicts NLOS on the KLT Dataset.

To empirically validate the accuracy of the deterministic geometric classifier, the framework’s predictions were cross-referenced against the publicly released KLT Dataset [9]. This open-source benchmark pairs raw GNSS observations with fisheye-camera-aided LOS/NLOS ground-truth labels collected in the deep urban canyons of Hong Kong. The results are shown in Figure 11.

The validation was executed on eight KLT sub-sequences featuring available ground truth, comprising 44,120 matched GNSS/camera samples. Out of these samples, the geometric model generated 4553 NLOS predictions. When compared against the camera-aided reference labels, 4326 of these predictions were true NLOS, yielding an NLOS precision of 95%. False NLOS predictions (227 instances) accounted for merely 0.51% of all matched samples. Statistically, when the framework declares a satellite as NLOS, it is correct in approximately 19 out of 20 cases, confirming that the vast majority of its NLOS detections correspond to genuine building obstructions.

Conversely, the NLOS recall was measured at 30.2%. Among the 14,343 ground-truth NLOS samples, 4326 were successfully detected by the framework, while 10,017 samples were incorrectly labeled as (LOS 69.8% missed). For the LOS class, the model achieved a precision of 74.7% (29,550 correct out of 39,567 geometric LOS predictions) and a high recall of 99.2% (29,550 out of 29,777 ground-truth LOS).

This statistical asymmetry provides a crucial operational insight into the framework’s behavior: the dominant error mode is false LOS rather than false NLOS. The geometric model is conservative in issuing NLOS labels, but trustworthy when it does. This behavior is directly attributable to the nature of the input data: while the 3D building mesh reliably captures macro-scale structural obstructions, it does not account for unmodeled physical blockers ubiquitous in real-world urban canyons, such as street trees, dense foliage, and large moving vehicles.

The KLT dataset provides binary camera-based LOS/NLOS ground-truth labels derived from fisheye immages. We conducted a qualitative sample check across the raw fisheye immages, corresponding to false LOS instances. This inspection confirmed our hypothesis: false LOS predictions occur when the satellite signal path is obstructed by real-world physical elements absent from static 3D OSM LoD1 building meshes, most notably dense roadside tree foliage.

Consequently, the ray-tracing output functions as a high-confidence, lower-bound estimator of true building-induced NLOS, and an optimistic, upper-bound enumerator of potential LOS receptions. This performance profile perfectly aligns with the framework’s intended role as a fast, scalable, first-pass analytical filter for urban GNSS availability upper-bound.

### 3.5. Computational Performance and Scalability Analysis

To quantitatively support the computational efficiency and scalability claims of the proposed framework, benchmark evaluations were conducted across both simulated scenarios.

As shown in Table 3, when evaluating the large-scale Genoa city model, the framework processed over 2.24 billion satellite rays in an end-to-end time of 18.25 min. The GPU-accelerated BVH early-exit ray-tracing core required only 162.11 s (sim 2.7 min), yielding a sustained processing throughput of 13.82 million rays/second. The performance breakdown reveals that non-GPU pre-processing (DEM elevation horizon extraction and orbit propagation) accounts for 81.5% (892.92 s) of the total time, confirming that the ray-tracing traversal algorithm is not the primary computational bottleneck. All evaluations were executed within a cloud-based Google Colab environment utilizing an NVIDIA T4 GPU (16 GB VRAM) paired with an Intel Xeon CPU.

Furthermore, on the Hong Kong KLT mesh comprising over $1.02 million triangles, the direct LOS kernel evaluated visibility in 0.37 s, demonstrating that the hierarchy partitioning scales effectively even when faced with ultra-dense geometric models.

## 4. Discussion

The results confirm that GNSS performance in urban environments is strongly influenced not only by the number of visible satellites, but also by their spatial distribution. In the historic center, the lower number of LOS satellites is associated with a substantial increase in both the mean HDOP and its variability compared with the coastal area. This indicates that dense urban canyons can generate highly variable satellite geometries, with significant changes in expected positioning quality even at nearby locations. This behavior is consistent with previous 3D-mapping-aided GNSS studies, where building geometry and satellite visibility are used to characterize or mitigate positioning degradation in urban environments [2,14].

The terrain ablation analysis also highlights that the relative importance of different obstruction sources depends on the local environment. While terrain accounts for only a negligible fraction of blocked satellite paths in the historic center, where nearby buildings dominate the local horizon, it becomes a major source of obstruction in the more open coastal area. This result shows that building-only models may be sufficient in dense urban canyons, whereas terrain information becomes important in areas characterized by significant surrounding relief. The inclusion of the DEM therefore improves the general applicability of the framework across different urban morphologies.

The KLT validation provides further insight into the behavior of the proposed geometric model. The high NLOS precision (95.0%) indicates that when the framework predicts an obstruction, this prediction is generally reliable. Conversely, the lower NLOS recall (30.2%), combined with the very high LOS recall (99.2%), shows that the main limitation is represented by obstacles that are not included in the 3D model. Inspection of the reference images indicates that vegetation and other non-modeled objects are responsible for many of these cases. Therefore, the generated LOS maps should be interpreted as estimates of structural visibility rather than as a complete description of the propagation environment.

This distinction is consistent with previous 3DMA GNSS approaches. Several studies use 3D city models to identify NLOS signals or to improve positioning through shadow matching and map-aided estimation [3,13,14]. More advanced methods additionally model reflection and diffraction effects [11]. In contrast, the objective of the present work is to generate computationally efficient city-scale visibility information rather than to reproduce the complete signal propagation process. The proposed framework can therefore identify persistent areas of poor GNSS geometry, while transient effects such as vegetation, traffic, multipath, and receiver-dependent errors remain outside the current model.

From an application perspective, these results suggest that the generated LOS and DOP maps could be used as a priori indicators of GNSS quality in autonomous navigation and Intelligent Transportation Systems. Road segments with persistently low visibility or poor satellite geometry could be identified before navigation and assigned a higher localization-risk cost during route planning. Such information is complementary to real-time GNSS measurements and multi-sensor navigation rather than a replacement for them. This extends previous GNSS-aware mapping approaches [4] by providing dense, precomputed visibility information over large road networks.

Finally, the computational results demonstrate that city-scale visibility analysis is feasible using GPU-accelerated ray tracing. Since most of the processing time is associated with terrain and geospatial preprocessing rather than with the LOS intersection stage itself, further optimization should primarily target DEM processing and reusable preprocessing operations. The main limitations remain the accuracy and completeness of the input 3D data and the absence of vegetation, reflection, and diffraction models. Future work will therefore focus on enriching the environmental representation and, most importantly, on validating the relationship between the predicted visibility maps and actual positioning errors along real vehicle trajectories.

## 5. Conclusions

This paper presented a scalable, automated 3D ray-tracing framework designed to predict GNSS signal availability and quality in complex urban environments. By integrating OpenStreetMap building geometries with Digital Elevation Models, the proposed methodology overcomes the limitations of flat-terrain models, providing a more realistic representation of both artificial structures and natural topographic occlusions.

While the current geometric approach inherently functions as an optimistic upper-bound estimator, due to the absence of unmodeled dynamic obstacles, urban vegetation, and physical RF propagation phenomena like diffraction, it provides immediate, actionable intelligence. Computational efficiency achieved through the integration of a CPU-built Bounding Volume Hierarchy and batched GPU execution allows GNSS assessment in a proactive way.

This framework offers value to the development of Intelligent Transportation Systems and autonomous vehicle routing. By providing detailed “a priori” maps of expected satellite visibility and the Dilution of Precision upper bound, the system enables the design of “GNSS-aware” navigation algorithms that can avoid structurally denied environments. Future work will focus on conducting a rigorous comparative benchmarking against other state-of-the-art 3DMA GNSS tools.

## Figures and Tables

**Table 1:** Statistical distribution of HDOP in two different environments.

Environment	Sample Size (n Coordinates)	HDOP Mean ± σ
Historic centre	3117	4.52±4.11
Coastal zone	1529	1.42±0.26

**Table 2:** Terrain ablation study across the Genoa sub-scenarios.

Sub-Scenario	Evaluation Points	Mean Satellites Blocked by Terrain	Occlusions Due to Terrain
Historic centre	3117	0.08±0.05	0.6%
Coastal zone	1529	1.54±0.42	51.3%
City-wide	171,711	1.21±0.68	14.2%

**Table 3:** Quantitative execution breakdown and throughput metrics for both experimental campaigns.

Metric	Genoa Area	Hong Kong KLT
Mesh triangles	460,320	102,633
BVH nodes	316,469	72,033
Wall-clock time	1095s (18.25min)	45.3s
Terrain preprocessing	892.9s	10s
BVH LOS kernel	162.1s	0.37s
LOS throughput	$13.82\times 10^{6}\;\mathrm{rays}/s$	$1.12\times 10^{4}\;\mathrm{rays}/s$
Platform	NVIDIA T4–Intel Xeon	NVIDIA T4–Intel Xeon

## Data Availability

The datasets and software code used in this study are openly available at the following locations: Source Code: The code used to generate the figures and run the ray-tracing pipeline is available at https://github.com/andrea444460/gnss_gpu_los_nlos (accessed on 10 June 2026). Terrain Elevation Data: Copernicus DEM is available via the Copernicus Data Space Ecosystem at https://dataspace.copernicus.eu/explore-data/data-collections/copernicus-contributing-missions/collections-description/COP-DEM (accessed on 10 June 2026). Building Footprints: OpenStreetMap vector data are accessible at https://www.openstreetmap.org (accessed on 10 June 2026).

## Abbreviations

The following abbreviations are used in this manuscript:

3DMA	3D Mapping-Aided
CUDA	Compute Unified Device Architecture
DEM	Digital Elevation Model
DOP	Dilution of Precision
DSM	Digital Surface Model
GNSS	Global Navigation Satellite System
GPU	Graphics Processing Unit
KLT	Kowloon Tong
LOS	Line-of-Sight
NLOS	Non-Line-of-Sight
NVIDIA	NVIDIA Corporation
OSM	OpenStreetMap
RTK	Real-Time Kinematic
SD	Standard Deviation
